# Sex- and Age-Specific Distribution of Skeletal Muscle Mass and Paraspinal Muscle Indices and Their Association With Spinal Sagittal Alignment: The Research on Osteoarthritis/Osteoporosis Against Disability (ROAD) Study

**DOI:** 10.7759/cureus.84972

**Published:** 2025-05-28

**Authors:** Naomi Iwane, Hiroshi Hashizume, Shizumasa Murata, Kanae Mure, Hiroyuki Oka, Toshiko Iidaka, Takahide Sasaki, Masatoshi Teraguchi, Keiji Nagata, Yuyu Ishimoto, Masanari Takami, Shunji Tsutsui, Hiroshi Iwasaki, Sakae Tanaka, Hiroshi Yamada, Noriko Yoshimura

**Affiliations:** 1 School of Health and Nursing Science, Wakayama Medical University, Wakayama, JPN; 2 Department of Orthopaedic Surgery, Wakayama Medical University, Wakayama, JPN; 3 Department of Public Health, Wakayama Medical University, Wakayama, JPN; 4 Division of Musculoskeletal AI System Development, Faculty of Medicine, The University of Tokyo, Tokyo, JPN; 5 Department of Preventive Medicine for Locomotive Organ Disorders, 22nd Century Medical and Research Center, The University of Tokyo, Tokyo, JPN; 6 Department of Orthopaedic Surgery, Faculty of Medicine, The University of Tokyo, Tokyo, JPN

**Keywords:** aging population, fat infiltration, paraspinal muscles, population-based cohort, sagittal spinal alignment, sarcopenia, spinal sagittal alignment

## Abstract

Background

Sagittal spinal malalignment is a significant contributor to reduced quality of life in older adults, associated with chronic back pain, impaired mobility, and psychosocial distress. While age-related loss of skeletal muscle mass (sarcopenia) is known to influence posture, the sex-specific progression of muscle degeneration and its association with sagittal balance remain unclear.

Objective

The objective of this study is to investigate the age- and sex-specific changes in skeletal and paraspinal muscle indices and their associations with sagittal spinal alignment in a large population-based Japanese cohort.

Methods

This cross-sectional study used data from the third visit of the Research on Osteoarthritis/Osteoporosis Against Disability (ROAD) study, including 748 community-dwelling adults (220 men, 528 women). Whole-spine lateral radiographs and lumbar magnetic resonance imaging were used to assess the C7 sagittal vertical axis (SVA) and paraspinal muscle composition, respectively. Skeletal muscle mass was evaluated using bioimpedance analysis. Participants were stratified into five age groups, and muscle quantity and quality were analyzed by sex. Multivariate regression analyses were performed to identify predictors of sagittal malalignment.

Results

As people age, both men and women show a clear increase in the forward tilt of the spine, measured by the C7 SVA, and an increase in fat within the deep back muscles, especially the multifidus and erector spinae. For example, the average SVA increased from 10.3 mm in those under 50 to 46.7 mm in those aged 80 and above (p < 0.001). The fat content in the multifidus at the L1/2 level rose from 12.4% to 29.6% in men and from 15.1% to 35.4% in women. Lower trunk muscle mass, rather than overall skeletal muscle or limb muscle index, was linked to worse spinal alignment in both sexes. Increased fat in the paraspinal muscles, especially at the L1/2 level, was more strongly related to poor posture in women.

Conclusion

Reduced trunk muscle mass and increased fat infiltration of paraspinal muscles, particularly at the L1/2 level, were independently associated with a forward-shifted SVA (SVA ≥50 mm), especially among women. These findings highlight the central role of trunk muscle quality and quantity in maintaining postural alignment in aging adults and suggest the importance of interventions targeting trunk musculature to prevent age-related sagittal imbalance.

## Introduction

In the context of a rapidly aging society, sagittal spinal imbalance has emerged as a critical health concern that significantly compromises the quality of life (QOL) among older adults [1]. As sagittal imbalance progresses, it has been identified as a major contributor to chronic low back pain [2,3]. Furthermore, recent evidence suggests that forward trunk inclination increases the risk of gastroesophageal reflux disease [4,5]. This forward-leaning posture may also negatively impact self-image, leading to loss of confidence in appearance, feelings of social isolation, and psychological distress such as shame and embarrassment [6].

The sagittal vertical axis (SVA) is a key radiographic parameter used to evaluate sagittal spinal alignment, reflecting the anteroposterior displacement of the body’s center of gravity relative to the pelvis during upright standing [7]. The SVA is measured in millimeters (mm) as the horizontal distance between a vertical plumb line drawn from the center of the seventh cervical vertebra (C7) and the posterosuperior corner of the sacrum (S1) on lateral radiographs. An SVA within ±50 mm is considered physiologically normal, whereas a forward deviation of more than +50 mm is commonly associated with chronic pain, muscle fatigue, impaired activities of daily living (ADL), and diminished QOL [7]. Accordingly, the SVA plays a pivotal role in the assessment of posture in older individuals and patients with spinal deformities, as well as in surgical decision-making and postoperative evaluation in spinal realignment procedures. Nevertheless, the underlying mechanisms and preventive strategies for the progression of sagittal malalignment remain insufficiently understood. Given the ethical responsibility inherent in studying aging populations, we conducted this research in strict accordance with ethical standards and obtained informed consent from all participants.

In recent years, increasing attention has been given to the impact of sarcopenia on postural stability among older adults. Sarcopenia is characterized by age-related declines in skeletal muscle mass and strength and is widely recognized as a contributor to impaired physical performance and heightened fall risk in elderly populations [8]. The appendicular skeletal muscle mass index (ASMI) is internationally accepted as a core diagnostic criterion for sarcopenia. Meanwhile, degenerative changes in spinal paraspinal muscles, such as the erector spinae and multifidus, have also been implicated in postural abnormalities and pain generation [9]. In particular, the fatty infiltration ratio (FIR; equivalent to FI%) of these muscles has attracted attention as an index reflecting qualitative changes in muscle tissue, and growing evidence links the FIR to both chronic low back pain and sagittal malalignment [10-12].

However, the longitudinal progression of age-related changes in appendicular and paraspinal muscle composition and the extent to which these changes influence postural alignment remain incompletely elucidated, especially regarding potential sex differences. The present study aims to provide new insights into this underexplored area.

The objectives of this investigation are twofold: (i) to clarify how degenerative changes in appendicular and paraspinal muscles progress with aging and (ii) to identify which muscle-related indices are most strongly associated with postural changes as reflected by the C7 SVA. These analyses were conducted using data from the third survey of the Research on Osteoarthritis/Osteoporosis Against Disability (ROAD) study.

While prior studies have demonstrated associations between paraspinal muscle degeneration and spinal alignment, most lacked sex-specific analyses or focused solely on small, clinical populations. The current study extends these findings by leveraging a large, community-based cohort to examine age- and sex-specific trajectories of muscle decline and their distinct relationships with sagittal imbalance.

## Materials and methods

Ethics approval

This study was approved by the Research Ethics Committee of the University of Tokyo (No. 1326). All participants provided written informed consent for the inclusion and publication of their data. All procedures were performed in accordance with the ethical standards of the responsible committee on human experimentation (institutional and national) and the Declaration of Helsinki.

Participants

**Figure 1:**
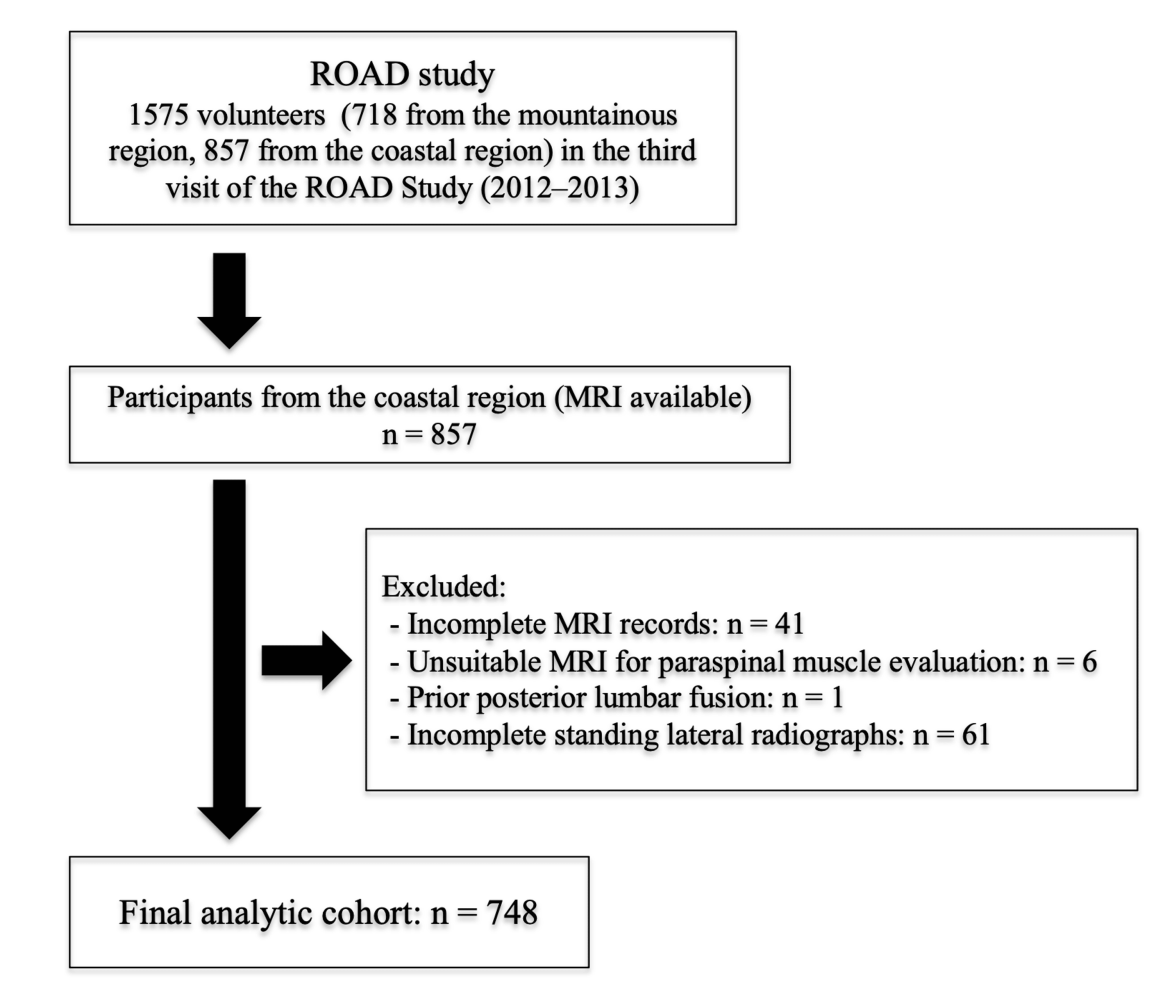
Flow chart of participant selection for cross-sectional analysis of paraspinal muscle evaluation and spinal alignment. Of the 1,575 participants enrolled in the third visit of the ROAD study (2012–2013), 857 individuals from the coastal region underwent MRI. After excluding those with incomplete MRI records (n = 41), unsuitable MRI for evaluating paraspinal muscles (n = 6), a history of posterior lumbar fusion (n = 1), and incomplete standing lateral radiographs of the whole spine (n = 61), 748 participants were included in the final analysis. ROAD: Research on Osteoarthritis/Osteoporosis Against Disability

The ROAD study is a prospective, population-based cohort initiated in three regions of Japan to investigate musculoskeletal health [13,14]. Of the 857 individuals originally recruited from coastal areas, 748 participants (220 men, 528 women) aged 40 to 89 years (mean age 63.6 ± 13.2 years) were included in the final analysis (Figure 1). These community-dwelling individuals were physically independent and capable of providing reliable health and lifestyle data. Participants were eligible if they resided in the community and were physically and cognitively capable of walking to the study site, providing accurate self-reported information, and signing informed consent. Comprehensive details of the study design and recruitment have been documented previously [13,14]. The present analysis utilized data from the third wave of the ROAD study, conducted between 2012 and 2013. During this phase, 1,575 participants were enrolled, 718 from a mountainous area and 857 from a coastal area. Due to budgetary constraints, MRI assessments were performed only for the coastal cohort. Consequently, the current investigation was limited to the 857 individuals from the coastal region. Exclusion criteria included 41 participants with incomplete MRI data, six individuals whose MRI images were inadequate for evaluating the paraspinal musculature, one patient with a history of posterior lumbar fusion surgery, and 61 participants lacking complete standing lateral whole-spine radiographs. These exclusions yielded the final analytic sample for this study.

Anthropometric measurements

Anthropometric measurements, including height and weight, were measured in all participants. Body mass index (BMI; weight [kg]/height [m^2^]) was calculated as weight in kilograms divided by height in meters squared.

Radiographic assessment

**Figure 2:**
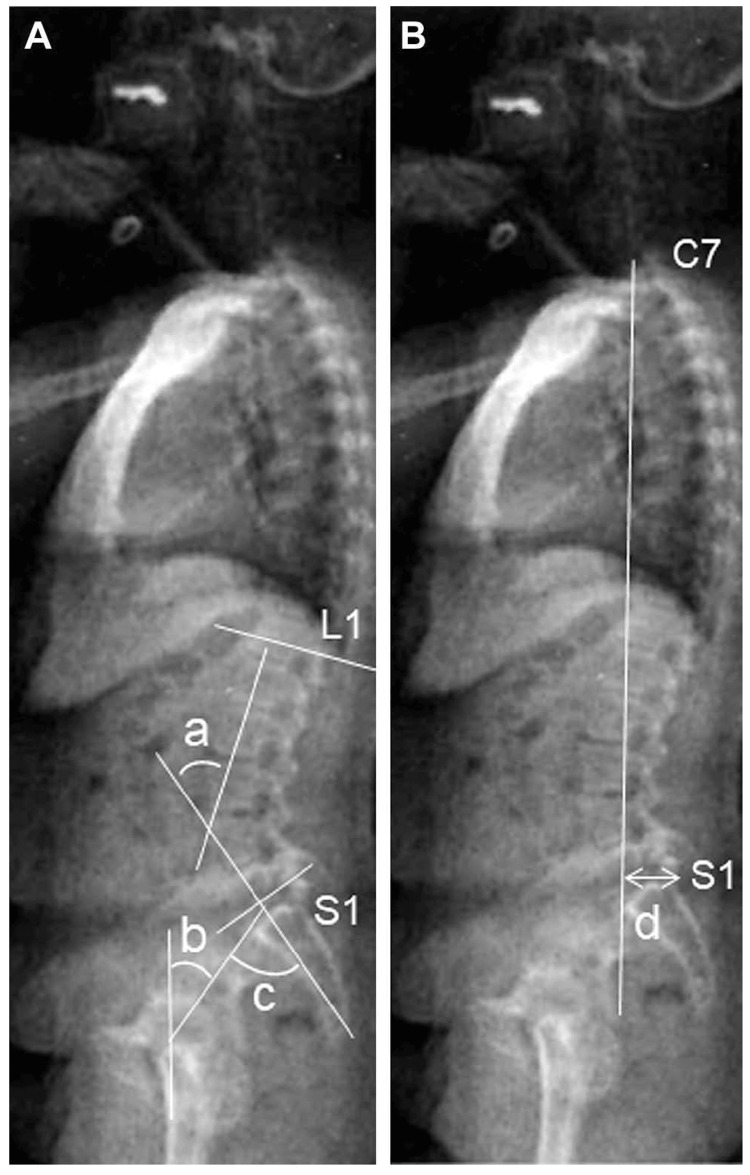
Radiographic assessment of sagittal spinal and pelvic parameters. Parameters measured on standing lateral radiographs of the whole spine and pelvis include: In panel A, (a) Lumbar lordosis: Cobb angle from the upper endplate of L1 to the lower endplate of S1; (b) Pelvic tilt: angle between the line connecting the midpoint of the sacral plate to the axis of the femoral heads and the vertical axis; (c) Pelvic incidence: angle between the line perpendicular to the sacral plate at its midpoint and the line connecting this point to the axis of the femoral heads; in panel B, (d) Sagittal vertical axis (SVA): horizontal distance from the C7 plumb line (originating at the middle of the C7 vertebral body) to the posterior superior endplate of S1. This figure was created using images and descriptions for which we retain the copyright, based on ref [15].

Standing lateral radiographs of the whole spine were obtained for each participant by licensed radiography technicians using a 40-inch film. Films were positioned so that the bones from C2 to the proximal femur were in the range. Each radiograph was aligned such that the film edge was the reference for vertical alignment. As described previously [15], participants were instructed to stand in a comfortable position with their hips and knees fully extended. The arms were flexed with the hands resting on supports at the level of the shoulders (Figure 2). The C7 SVA was measured on lateral radiographs as the horizontal distance from the C7 plumb line originating at the middle of the C7 vertebral body to the posterior superior endplate of S1 [16-18].

Magnetic resonance imaging

A mobile MRI unit (Achieva 1.5 T; Philips Medical Systems, Best, the Netherlands) was used, and whole-spine MRI was performed for all participants on the same day as the examination [19]. The participants were supine during the MRI, and those with rounded backs used triangular pillows under their heads and knees. MRI was performed using sagittal and axial T2-weighted fast spin echo sequences. Sagittal images covered the entire spine, while axial images were acquired at each lumbar intervertebral level (T12/L1 to L5/S1) in planes parallel to the vertebral endplates. These axial images were used to assess paraspinal muscle size and fat content using established measurement techniques.

Measurement of the cross-sectional area and FIR of paraspinal muscles

**Figure 3:**
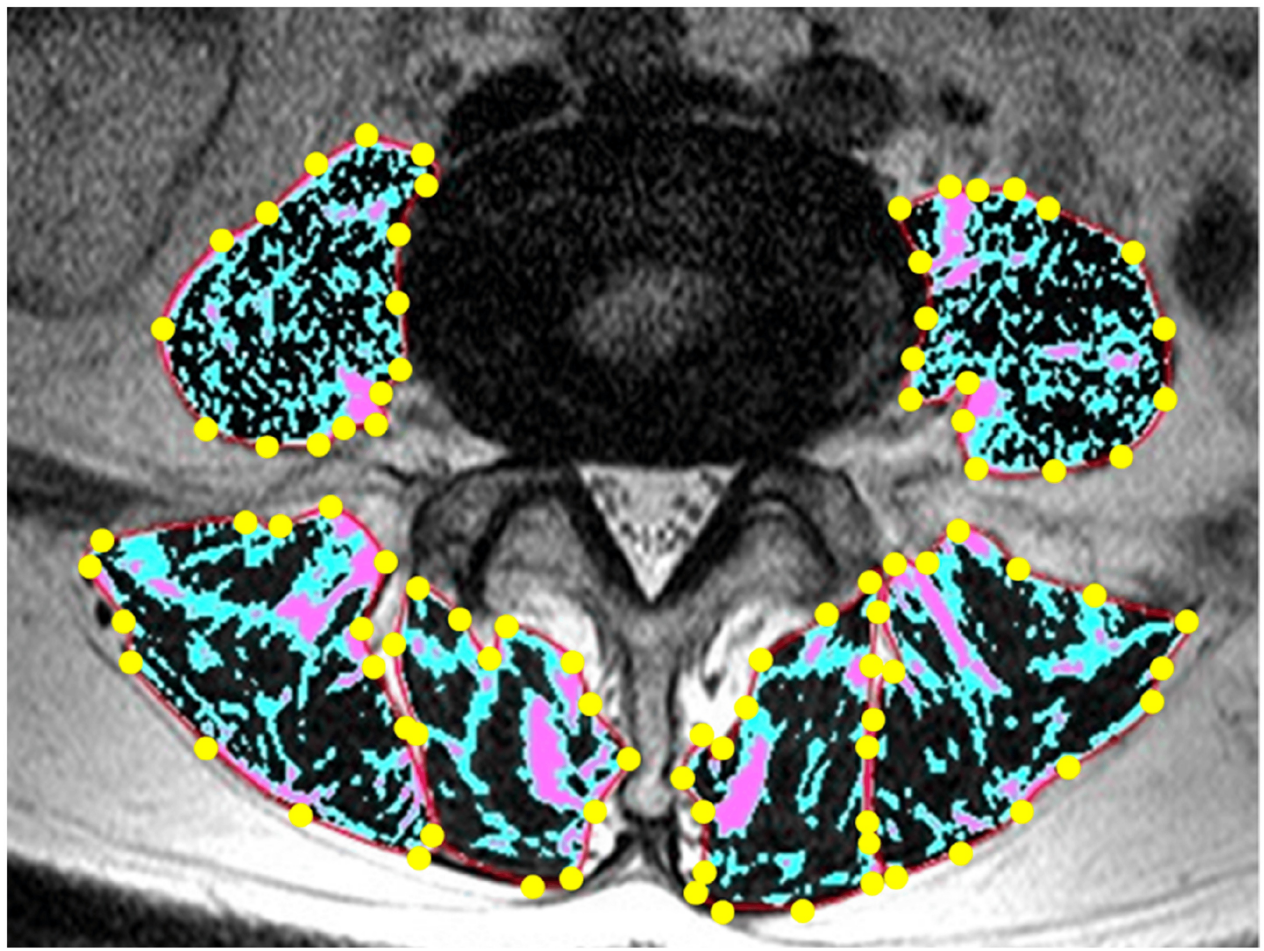
Measurement technique for the cross-sectional area (CSA) and fatty infiltration ratio (FIR) of paraspinal muscles. The regions shown in black, blue, and red represent areas of low, medium, and high signal intensity, respectively. The high-intensity area was defined as the fatty infiltration area. This figure was created using images and descriptions for which we retain the copyright, based on ref [19].

Quantitative evaluation of the paraspinal muscles, including the erector spinae, multifidus, and psoas major, was conducted using axial T2-weighted MRI scans analyzed on a radiological workstation optimized for musculoskeletal assessment. The cross-sectional area (CSA) and FIR were measured for the erector spinae and multifidus at spinal levels T12/L1 through L4/5, and for the psoas major specifically at the L4/5 level (Figure 3) [19]. The CSA was determined by manually tracing the outer contour of each target muscle to construct a polygonal region of interest. The FIR was defined as the proportion of the area occupied by fatty tissue, calculated by dividing the fatty infiltration area by the total muscle area. While the FIR was measured at all lumbar levels from T12/L1 to L4/5, we selected L1/2 and L4/5 as representative sites for reporting the results. These levels were chosen to reflect both the upper and lower lumbar spine, based on their clinical relevance and to enhance clarity by avoiding redundancy. The age-related trends in FIR at these levels were consistent with those observed at intermediate levels. Fatty regions within the muscle were identified using a clustering method that classified all pixels in the region of interest into three distinct groups based on signal intensity. To assess the reliability of the CSA and FIR measurements, intraclass correlation coefficients (ICCs) were calculated. For interobserver reliability, 80 MRI scans were randomly selected and independently analyzed by two orthopedic surgeons (TS and HI). Intraobserver reliability was evaluated by repeating measurements on the same images by one observer (TS) at an interval of more than one month. All ICC values for both the CSA and FIR were 0.99, indicating excellent reproducibility.

Skeletal muscle mass

Skeletal muscle mass was measured by bioelectrical impedance analysis (BIA) [20-23] using the Body Composition Analyzer MC-190 (Tanita Corp., Tokyo, Japan). The protocol was described by Tanimoto et al. [24,25] and has been validated previously [26]. Appendicular skeletal muscle mass (ASM) was calculated as the sum of the muscle mass of the arms and legs. Absolute ASM was converted to an appendicular muscle mass index by dividing by height in meters squared (kg/m^2^).

Statistical analysis

Descriptive statistics were determined and are presented as mean and standard deviation or frequency and percentage, unless otherwise specified. Differences in proportions were examined using the chi-square test, or Fisher’s exact test when the expected cell size was < 5. For continuous variables, Student’s t-test was used following confirmation of normality via the Shapiro-Wilk test. In observing the changes in the C7 SVA and muscle indicators due to aging, we divided the participants into five age groups, under 50, 50s, 60s, 70s, and over 80, then observed the changes in each measurement item by gender and age group, and performed a Dunnett's test with those under 50 as the standard. Next, we performed multiple regression analysis by gender, with C7 SVA as the objective variable and age, ASMI, erector spinae FIR, and multifidus FIR as the explanatory variables [27]. All statistical analyses were performed using JMP Pro version 16 (SAS Inc., Cary, NC, USA). A P-value<0.05 was set to indicate statistical significance. Dunnett’s test was used to compare age groups with a uniform control (<50 years), minimizing type I error due to multiple comparisons. Multivariable regression was chosen to adjust for key covariates such as age and BMI, enabling robust identification of independent associations.

Data availability

The datasets generated and/or analyzed during the current study are available only to approved co-investigators of the ROAD Study, in accordance with the data sharing policy jointly defined by the Research Ethics Committee of the University of Tokyo.

Requests for data access may be considered on a case-by-case basis, subject to approval by the study steering committee. The data are not publicly available due to ethical and privacy restrictions.

## Results

Characteristics of the participants

The baseline characteristics of the 748 participants are summarized in Table 1. Overall, the cohort included 220 men and 528 women, with a mean age of 63.6 ± 13.2 years. Further stratified comparisons across age groups are shown in Table 2.

**Table 1:** Baseline characteristics of the participants. Data are presented as mean ± standard deviation (SD) for continuous variables and as number (percentage) for categorical variables.

	Men	Women
Number of participants	220	528
Age (years)	62.9±14.0	63.8±12.7
Age strata (number)		
<50	36(16.4)	66(12.5)
50-59	43(19.6)	113(21.4)
60-69	74(33.6)	159(30.1)
70-79	38(17.3)	140(26.5)
≧80	29(13.2)	50(9.5)
Height (cm)	166.7±6.7	153.2±6.4
Weight (kg)	66.6±10.9	53.0±8.9
Body mass index (kg/m^2^)	23.9±3.5	22.6±3.6

**Table 2:** Representative values by gender and age group for each evaluated indicator. All data are shown as mean [95% confidence interval]. SVA: sagittal vertical axis, SMI: skeletal muscle mass index, TMI: trunk muscle mass index, FIR: fatty infiltration ratio, MF: multifidus, ES: erector spinae. Dunnett's test was performed using 50 years of age as the standard for both genders. Statistically significant p-values (p < 0.05) are indicated in bold. *p<0.05, **p<0.01, ***p<0.001

	<50	50-59	60-69	70-79	≧80
Men					
Height (cm)	172.2[169.8-174.6]	168.4[166.4-170.4]*	166.7[165.6-167.8]***	163.6[161.6-165.6]***	161.2[159.1-163.3]***
Weight (kg)	72.8[69.4-76.2]	67.6[64.1-71.1]	67.465.3-69.6]*	65.5[62.0-69.0]**	56.3[53.5-59.1]***
BMI (kg/m^2^)	24.6[23.4-25.9]	23.8[22.8-24.8]	24.3[23.5-25.1]	24.4[23.2-25.5]	21.7[20.5-23.0]**
C7 SVA (cm)	-11.1[-18.2--3.9]	5.7[-3.3-14.7]	6.8[-1.1-14.7]	26.3[9.9-42.8]***	33.5[17.6-49.5]***
Skeletal muscle mass (kg)	25.8[24.9-26.7]	23.3[22.2-24.2]**	22.6[22.0-23.2]***	21.1[20.0-22.2]***	18.0[17.1-18.8]***
SMI (kg/m^2^)	8.7[8.4-9.1]	8.2[7.9-8.5]	8.1[7.9-8.3]*	7.9[7.5-8.2]**	6.9[6.6-7.2]***
Trunk muscle mass (kg)	28.9[28.1-29.8]	27.8[27.0-28.7]	27.2[26.8-27.6]**	25.9[25.1-26.6]***	24.2[23.0-25.3]***
TMI (kg/m^2^)	9.8[9.5-1.0]	9.8[9.6-1.0]	9.8[9.6-9.9]	9.7[9.5-9.9]	9.4[8.9-9.8]
FIR of MF at L1/2 level (%)	6.1[5.4-6.7]	7.4[6.6-8.3]	8.3[7.8-8.9]**	11.9[10.5-13.3]***	13.4[11.7-15.1]***
FIR of ES at the L1/2 level (%)	5.0[4.1-5.9]	5.4[4.9-5.8]	5.9[5.5-6.3]	8.3[7.0-9.5]***	7.9[6.9-8.9]***
FIR of MF at the L4/5 level (%)	8.0[6.4-9.5]	10.0[7.9-12.1]	9.3[8.3-10.3]	12.0[10.1-13.8]**	13.3[11.1-15.6]***
FIR of ES at the L4/5 level (%)	9.2[7.5-10.9]	9.7[8.6-10.8]	10.2[9.4-11.0]	12.0[10.8-13.2]**	13.7[12.4-15.0]***
Women					
Height (cm)	158.6[157.5-159.7]	155.6[154.7-156.4]**	153.2[152.3-154.2]***	150.9[149.9-151.9]***	147.0[145.4-148.6]***
Weight (kg)	53.2[51.0-55.3]	55.3[53.5-57.0]	52.7[51.3-54.0]	53.1[51.7-54.5]	48.9[46.7-51.0]*
BMI (kg/m^2^)	21.1[20.2-22.0]	22.9[22.2-23.5]**	22.4[21.9-23.0]*	23.3[22.7-23.9]***	22.6[21.6-23.6]
C7 SVA (cm)	-15.2[-20.7--9.6]	-4.7[-9.1--0.3]	3.0[-1.2-7.2]**	23.2[16.5-29.8]***	63.3[25.5-81.3]***
Skeletal muscle mass (kg)	16.3[15.9-16.7]	15.6[15.3-16.0]*	14.5[14.2-14.7]***	13.9[13.7-14.2]***	12.8[12.3-13.3]***
SMI (kg/m^2^)	6.5[6.4-6.6]	6.5[6.3-6.6]	6.2[6.1-6.3]**	6.1[6.0-6.2]***	5.9[5.7-6.1]***
Trunk muscle mass (kg)	20.1[19.6-20.5]	20.0[19.7-20.2]	19.7[19.4-19.9]	19.4[19.1-19.7]*	18.6[18.2-19.1]***
TMI (kg/m^2^)	8.0[7.8-8.1]	8.3[8.2-8.3]***	8.4[8.3-8.4]***	8.5[8.4-8.6]***	8.6[8.5-8.8]***
FIR of MF at the L1/2 level (%)	7.5[7.0-8.1]	9.9[9.3-10.5]***	11.6[11.0-12.2]***	13.8[13.0-14.5]***	17.3[15.6-18.9]***
FIR of ES at the L1/2 level (%)	5.5[5.2-5.8]	6.5[6.2-6.9]	7.7[7.3-8.1]***	9.7[9.1-10.3]***	11.8[10.0-13.5]***
FIR of MF at the L4/5 level (%)	9.6[8.6-10.7]	11.0[10.2-11.9]	12.8[11.9-13.6]**	14.9[13.8-16.0]***	17.9[15.3-20.6]***
FIR of ES at the L4/5 level (%)	10.2[9.2-11.2]	11.0[10.3-11.7]	12.5[11.9-13.1]**	14.1[13.3-15.0]***	15.9[14.1-17.8]***

Distribution of C7 SVA and muscle indices among men and women

Participants were stratified into five age groups: <50, 50s, 60s, 70s, and ≥80 years. Dunnett’s test was performed using the <50 group as the reference.

In men, the C7 SVA increased significantly with age, with statistical significance observed in the 70s and ≥80 groups. Skeletal muscle mass declined significantly beginning in the 50s (p < 0.05), and the skeletal muscle mass index (SMI) showed significant decreases from the 60s onward. Trunk muscle mass also decreased significantly from the 60s, with further reductions in the 70s and ≥80 groups. In contrast, the trunk muscle mass index (TMI) remained stable across all age groups, with no significant change.

FIRs of the multifidus and erector spinae muscles increased significantly with age at both the L1/2 and L4/5 levels. The FIR of the multifidus at the L1/2 level showed significant increases from the 60s onward, while significant elevations at the other sites were observed from the 70s. The highest FIR values were seen in the ≥80 group.

In women, the C7 SVA increased significantly with age, with statistically higher values observed from the 60s onward. Skeletal muscle mass showed significant reductions beginning in the 50s, and the SMI declined significantly from the 60s. Trunk muscle mass decreased significantly in the 70s and became more pronounced in the ≥80 group. In contrast, the TMI exhibited a slight but statistically significant increase with age.

The FIR of the multifidus at the L1/2 level increased significantly starting in the 50s, while the FIR of the erector spinae at the same level, as well as both muscles at the L4/5 level, showed significant increases from the 60s onward. FIR values progressively increased with age, with the most marked changes observed in the 70s and ≥80 groups.

Associations between muscle indices and sagittal alignment

To evaluate the relationship between spinal sagittal alignment and various muscle indices, we conducted multivariable linear and logistic regression analyses using the C7 SVA as the outcome. All models were adjusted for age and body mass index (BMI).

Linear regression analysis

In models treating the C7 SVA as a continuous variable, trunk muscle mass and the TMI were significantly and inversely associated with sagittal imbalance in men, whereas skeletal muscle mass and the SMI were not. In women, both skeletal muscle mass and trunk muscle mass showed significant inverse associations, while the SMI and TMI were not associated (Table 3).

**Table 3:** Multivariable linear regression analysis of factors associated with the sagittal vertical axis. SVA: sagittal vertical axis; SMI: skeletal muscle mass index (kg/m²); TMI: trunk muscle mass index (kg/m²); FIR: fatty infiltration ratio (%); PM: psoas major; ESM: erector spinae muscle. All models were adjusted for age and body mass index. Statistically significant p-values (p < 0.05) are indicated in bold.

	Explanation variable	Standard β	p-value
Men			
Model 1	Skeletal muscle mass	0.02	0.8426
Model 2	SMI	-0.05	0.7538
Model 3	Trunk muscle mass	-0.19	0.0149
Model 4	TMI	-0.34	<0.0001
Model 5	FIR of MF at the L1/2 level	0.32	<0.0001
Model 6	FIR of ES at the L1/2 level	0.40	<0.0001
Model 7	FIR of MF at the L4/5 level	0.16	0.0125
Model 8	FIR of ES at the L4/5 level	0.07	0.2681
Women			
Model 1	Skeletal muscle mass	-0.15	0.0089
Model 2	SMI	-0.05	0.4416
Model 3	Trunk muscle mass	-0.10	0.0118
Model 4	TMI	0.00	0.9710
Model 5	FIR of MF at the L1/2 level	0.22	<0.0001
Model 6	FIR of ES at the L1/2 level	0.24	<0.0001
Model 7	FIR of MF at the L4/5 level	0.18	<0.0001
Model 8	FIR of ES at the L4/5 level	0.18	<0.0001

As for qualitative measures, FIRs of the paraspinal muscles at both the L1/2 and L4/5 levels were positively associated with the C7 SVA in both sexes. In men, the FIR at the L1/2 level, particularly in the erector spinae, demonstrated the strongest associations, while the FIR at the L4/5 level showed less consistent results. In contrast, all FIR indices were significantly associated with sagittal alignment in women.

Logistic regression analysis

When examining sagittal malalignment as a binary outcome (C7 SVA ≥ 50 mm), trunk muscle mass was the only quantitative index consistently associated with malalignment in both men and women (Table 4). This finding supports earlier research emphasizing the central role of trunk musculature, especially the lumbar extensors, in maintaining upright posture during aging [9,10]. Skeletal muscle mass and the SMI were not significantly related to malalignment risk.

**Table 4:** Multivariable logistic regression analysis of factors associated with sagittal malalignment (C7 SVA ≥ 50 mm). SVA: sagittal vertical axis; SMI: skeletal muscle mass index (kg/m²); TMI: trunk muscle mass index (kg/m²); FIR: fatty infiltration ratio (%); PM: psoas major; ESM: erector spinae muscle; AUC: area under the curve. All models were adjusted for age and body mass index. Statistically significant p-values (p < 0.05) are indicated in bold.

	Explanation variable	Unit	Odds ratio	95% confidence interval	p-value	AUC
Men						
Model 1	Skeletal muscle mass	-1kg	1.03	0.82-1.28	0.8202	0.70
Model 2	SMI	-1kg/m^2^	1.07	0.46-2.58	0.8701	0.70
Model 3	Trunk muscle mass	-1kg	1.27	1.04-1.56	0.0183	0.73
Model 4	TMI	-1kg/m^2^	2.80	1.41-5.99	0.0030	0.76
Model 5	FIR of PM at the L1/2 level	+1%	1.18	1.05-1.32	0.0040	0.79
Model 6	FIR of ES at the L1/2 level	+1%	1.24	1.06-1.43	0.0040	0.76
Model 7	FIR of PM at the L4/5 level	+1%	1.07	1.00-1.14	0.0644	0.73
Model 8	FIR of ES at the L4/5 level	+1%	0.98	0.86-1.10	0.6978	0.70
Women						
Model 1	Skeletal muscle mass	-1kg	1.15	0.92-1.43	0.2093	0.82
Model 2	SMI	-1kg/m^2^	1.38	0.68-2.80	0.3711	0.82
Model 3	Trunk muscle mass	-1kg	1.08	0.92-1.27	0.3765	0.81
Model 4	TMI	-1kg/m^2^	1.08	0.50-1.70	0.7932	0.81
Model 5	FIR of PM at the L1/2 level	+1%	1.16	1.09-1.25	<0.0001	0.85
Model 6	FIR of ES at the L1/2 level	+1%	1.24	1.12-1.38	<0.0001	0.85
Model 7	FIR of PM at the L4/5 level	+1%	1.07	1.03-1.12	0.0008	0.83
Model 8	FIR of ES at the L4/5 level	+1%	1.07	1.01-1.13	0.0158	0.83

Regarding qualitative indices, the FIR of the paraspinal muscles at the L1/2 level was significantly associated with malalignment in both sexes. In women, the FIR at both L1/2 and L4/5 levels was consistently associated with increased odds of malalignment, while in men, the association was more evident at the L1/2 level. This is in line with previous studies that reported a relationship between fat infiltration in the lumbar extensors and global sagittal imbalance in older adults [10,11].

## Discussion

This study investigated age- and sex-specific changes in skeletal muscle indices and their relationship with sagittal spinal alignment in a general Japanese population. Using data from the third survey of the ROAD study, we found that both ASM and paraspinal muscle quality, particularly the FIR of the multifidus and erector spinae, underwent progressive, sex-dependent changes with aging. Moreover, these degenerative muscle parameters showed differential associations with sagittal imbalance, as measured by the C7 SVA, between men and women.

The present cross-sectional analysis delineates the trajectory of age-related alterations in spinal sagittal alignment and trunk musculature among community-dwelling adults. Consistent with prior epidemiological and biomechanical investigations [28,29], a progressive increase in the C7 SVA was observed with advancing age in both sexes, accompanied by significant declines in skeletal muscle mass, SMI, and trunk muscle mass. These findings reinforce the notion that degenerative changes in the musculoskeletal system are intricately linked to the development of sagittal malalignment in older populations. A salient and novel finding of this study pertains to the sex-specific divergence in the age-related behavior of the TMI, defined as trunk muscle mass adjusted for height squared. While trunk muscle mass declined significantly in both sexes, the TMI remained stable in men yet paradoxically increased in women. This discrepancy is attributable to the more pronounced loss of stature observed in aging women, likely due to vertebral compression, thoracic kyphosis, and disc degeneration [28]. As the TMI is sensitive to reductions in height, the shrinking denominator may offset or surpass the reduction in muscle mass, leading to an artifactual elevation of the index. This highlights the interpretive limitations of height-normalized indices in elderly cohorts and suggests the need for cautious interpretation, particularly in female populations. Moreover, qualitative deterioration of muscle, reflected by the FIR of the paraspinal muscles, exhibited a marked age-related increase at both the L1/2 and L4/5 levels. FIR elevations were evident from the 60s onward in most regions and were especially prominent in the ≥70-year female groups. Deep spinal extensors are essential for keeping the body upright. An increase in FIR may weaken these muscles and lead to worsening of sagittal imbalance [29]. Prior studies have demonstrated that increased intramuscular fat is associated with impaired muscle function and reduced spinal support capacity [8,30], underscoring the relevance of muscle quality as a determinant of spinal alignment. Among the muscle indices assessed, trunk muscle mass demonstrated the strongest and most consistent association with the C7 SVA, as confirmed by both linear regression analysis and logistic regression modeling for sagittal malalignment. Neither skeletal muscle mass nor SMI was significantly correlated with SVA. This is anatomically and biomechanically plausible, given that the trunk muscles-particularly the paraspinal extensors-play a direct role in resisting anterior trunk inclination [29]. These findings suggest that trunk muscle mass may serve as a more functionally relevant and clinically useful indicator of spinal alignment risk than generalized or limb-based muscle indices. It should be noted, however, that similar C7 SVA values can arise from distinct patterns of regional malalignment, such as cervical kyphosis or thoracolumbar deformities. These variations likely impose different biomechanical demands on segment-specific trunk muscles, particularly the cervical extensors versus the lumbar and thoracic paraspinals. While our study used C7 SVA as a global indicator of sagittal alignment, incorporating regional spinopelvic parameters in future research may provide a more comprehensive understanding of postural compensation and muscle burden. Several limitations of this study must be acknowledged. First, the cross-sectional design precludes inference of causality between muscle degeneration and spinal malalignment. Longitudinal data are required to elucidate temporal relationships. Second, muscle quantity and quality were assessed using BIA and MRI-derived fat infiltration, respectively; while widely accepted, BIA may be influenced by hydration status and postural variation. Third, the study population comprised ambulatory, community-dwelling adults, potentially introducing healthy volunteer bias and limiting extrapolation to frailer or institutionalized individuals. Additionally, although age and sex were considered, residual confounding by unmeasured variables such as physical activity, nutrition, or spinal pathology cannot be excluded. These limitations may have led to underestimation or overestimation of muscle-related effects on sagittal alignment. For example, the cross-sectional design prevents causal inference, and BIA may be influenced by hydration status. Future studies using longitudinal follow-up and MRI-based volumetric muscle analysis may help validate and expand upon these findings. While the methodological framework, including the use of standardized indices and validated imaging parameters, enhances the reproducibility of this study, its generalizability may be limited. The results reflect a relatively healthy Japanese cohort, and inter-population differences in height loss trajectories, musculoskeletal aging, and lifestyle may impact the external validity of the findings. A recent meta-analysis emphasized the variability in sarcopenia prevalence across global populations depending on measurement protocols and ethnic backgrounds [31]. Future multicenter, multiethnic longitudinal studies are warranted to determine whether the observed associations are consistent across diverse clinical and demographic settings.

In addition, future studies should evaluate regional sagittal alignment parameters such as cervical curvature, thoracic kyphosis, and lumbar lordosis to better understand the distribution of muscle burden. This is particularly important given our finding that similar SVA values may result from different deformity patterns, each likely requiring different compensatory muscular activation.

## Conclusions

This study highlights the central role of trunk muscle mass and quality in age-related changes in spinal alignment, while cautioning against overreliance on height-adjusted indices such as TMI, particularly in elderly women. Trunk muscle mass emerges as a robust and clinically meaningful marker of sagittal imbalance risk.

Clinically, these findings support the utility of incorporating trunk muscle assessment into routine musculoskeletal evaluations in older adults. Moreover, preventive or rehabilitative interventions, such as targeted trunk extensor strengthening programs, may be valuable in preserving postural alignment and preventing functional decline. Interventions aimed at preserving or enhancing trunk musculature may prove pivotal in mitigating age-associated postural decline and related health outcomes.
